# Effects of ultrasound-guided stellate ganglion block on cervical vascular blood flow: study protocol for a randomized controlled trial

**DOI:** 10.1186/s13063-018-2736-y

**Published:** 2018-08-07

**Authors:** Shaofeng Pu, Jie Chen, Xing Gu, Yongming Xu, Junzhen Wu, Yingying Lv, Dongping Du

**Affiliations:** 1 Department of Pain Management, Shanghai Jiao Tong University Affiliated Sixth People’s Hospital, 600 Yi Shan Rd, Shanghai, 200233 People’s Republic of China; 2 Department of Ultrasound in Medicine, Shanghai Jiao Tong University Affiliated Sixth People’s Hospital, Shanghai Institute of Ultrasound in Medicine, Shanghai, 200233 China; 3 Department of Gynaecology and Obstetrics, the Affiliated Kunshan First People’s Hospital, Jiangsu University, Kunshan, 215300 China

**Keywords:** Stellate ganglion block, Blood flow, Primary headaches, Randomized control crossover trial

## Abstract

**Background:**

The stellate ganglion block (SGB) can lead to vasodilation of the head and neck. However, controversy remains concerning the changes in extracerebral blood flow. The objective of this study is to assess the effects of SGB on the blood flow to the neck.

**Methods:**

A randomized controlled crossover trial with 38 participants will be conducted. Participants who have primary headaches will be assigned to either group A or B. Patients in group A will receive SGB with 6 ml 1% lidocaine, and after a one-week washout period, they will undergo the second SGB with 6 ml normal saline. In contrast, patients in group B will receive the opposite protocol. Data will be collected at baseline (T0) and at 15 min after the first intervention (T1), 15 min before the second intervention (T2), 15 min after the second intervention (T3) and at a 3-week follow up (T4). T1 is the primary time point for the primary outcome analysis. The primary outcomes include the peak systolic velocity (PSV), the end diastolic velocity (EDV), resistance index (RI) and vessel diameter of the common carotid artery (CCA) and vertebral artery (VA). The secondary outcomes include the rate of ptosis, the rate of conjunctival flushing, and the numerical rating scale (NRS) pain score. Additionally, adverse events (AEs) or serious adverse events (SAEs) will be collected at each assessment point.

**Discussion:**

This study will comprehensively investigate the efficacy of SGB in extracerebral blood flow. Our research may also suggest that SGB will be effective in reducing pain in patients with primary headaches.

**Trial registration:**

Chinese Clinical Trial Registry, identifier ChiCTR-IOR-17011536. Registered on 1 June 2017.

**Electronic supplementary material:**

The online version of this article (10.1186/s13063-018-2736-y) contains supplementary material, which is available to authorized users.

## Background

The stellate ganglion measures approximately 2.5 cm in length, 1 cm in width, and 0.5 cm in thickness. It is located posteriorly in the chest, in front of the neck of the first rib, and may extend to the seventh cervical (C7) vertebral body [1]. The stellate ganglion block (SGB) is an accepted intervention for the treatment of a variety of pain conditions of the head and neck regions as well as the upper limbs [2]. SGB is also effective in the treatment of phantom pain, postherpetic neuralgia, cancer pain, cardiac arrhythmias, orofacial pain, and vascular headache [3].

SGB is conventionally performed using a blind technique. However, this blind technique can cause various adverse effects, such as inadvertent epidural, subarachnoid, or intravascular injection, formation of haematomas, and oesophageal injury [4–6]. Ultrasound-guided SGB was introduced in 1995 [7]. Ultrasound scanning can allow for imaging and distinction of the anatomical structure of the neck. Ultrasound-guided SGB is safer than the conventional technique and allows the use of a small injectate volume while maintaining the same degree of efficacy.

SGB leads to vasodilation of the head and neck. However, controversy remains regarding the changes in cerebral and extracerebral blood flow in the head. Liu et al. [8] reported that the blood flow velocities of the internal carotid artery (ICA) on the ischaemic side were decreased and the resistance indexes were increased in 12 patients with ischaemic optic neuropathy (ION) after daily SGB treatment on the affected side with 2–3 mL of 2% lidocaine, for a treatment period of 10–15 days. In contrast, the blood flow velocities of the ICA on the ischaemic side were increased and the resistance indexes were decreased. Ohinata et al. [9] reported that patients who underwent SGB experience increased blood flow of the common carotid artery (CCA) and vertebral artery (VA) on the side of the SGB and decreased blood flow on the opposite side. Nitahara et al. [10] showed that SGBs with 6–8 mL of 1% mepivacaine can significantly increase the blood flow velocity in the CCA, whereas velocity in the VA was unchanged. On the side contralateral to the SGB, significant changes in blood flow velocity in the CCA and VA were never observed. SGB can increase blood flow in the anastomotic artery after superficial temporal artery-middle cerebral artery bypass [11]. In patients with subarachnoid haemorrhage (SAH) after SGB, a significant increase in the calibre of the middle cerebral artery (MCA), VA, and arteriae basilaris (BA) was observed. It has also been shown that the calibre of the MCA, VA, and BA was increased in patients without SAH after treatment with SGB [12].

Kapral et al. [7] and Narouze et al. [13] reported a successful treatment effect with 5 mL of local anaesthetic in ultrasound-guided SGB. Two studies reported on the optimal volume of local anaesthetic required for successful ultrasound-guided SGB compared to the traditional approach [14, 15]. One study showed that 4 ml of 0.2% ropivacaine was sufficient for a successful block [14], while the other showed the optimal volume was 2 ml of 0.5% mepivacaine [15].

Therefore, there is no strong evidence regarding the effect of SGB on the blood flow of the CCA and VA. The blocking effect may be different due to the different anaesthetic drugs, the different volume, or the number of treatments used in SGB. Our aim was to design and conduct a double-blind, randomized controlled crossover trial to evaluate the effects of ultrasound-guided SGB on the CCA and VA in subjects with primary headaches. We chose to use 6 ml 1% lidocaine to perform the SGB because lidocaine is safe and this injection volume can reliably ensure the success of SGB.

### Objective

We designed this prospective, double-blinded, controlled crossover study to investigate the cervical blood flow changes after SGB. The peak systolic velocity (PSV), the end diastolic velocity (EDV), diameter, and resistance index (RI) of the CCA and VA will be measured before and 15 min after each procedure. The rate of ptosis, conjunctival flushing, and the pain scores will also be recorded.

### Trial design

A double-blinded, randomized controlled crossover trial will be conducted, and all subjects will undergo ultrasound-guided SBG. Group A will first undergo SGB with 6 ml 1% lidocaine. After a one-week washout period, this group will receive ultrasound-guided SGB with 6 ml normal saline. In contrast, group B will receive the opposite protocol. That is, group B subjects will first receive SGB with 6 ml normal saline, and after a one-week washout period, this group will receive SGB with 6 ml 1% lidocaine.

## Methods

### Setting

**Fig. 1:**
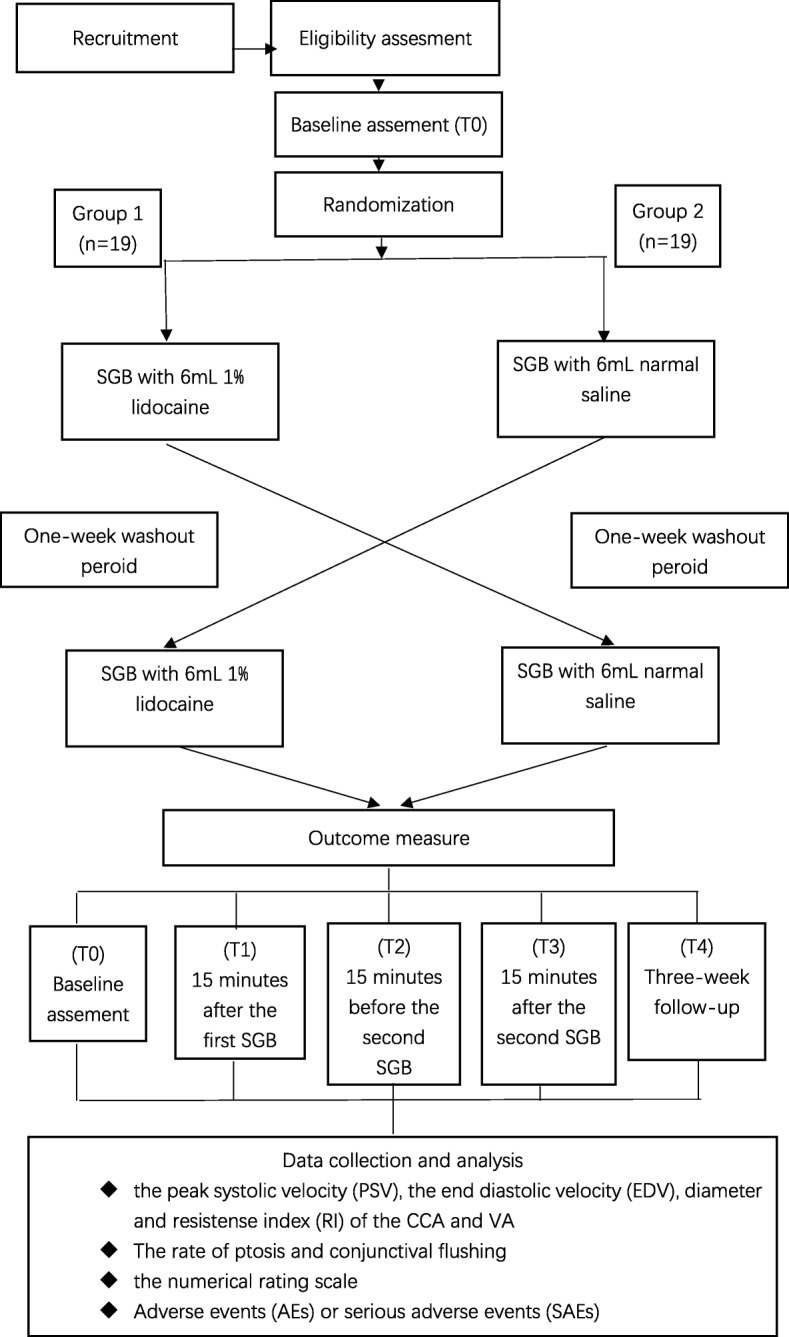
Participant flow diagram according to Consolidated Standards of Reporting Trials (CONSORT)

This trial is a prospective, investigator and observer-blinded, randomized, crossover trial. This study will be carried out in the Shanghai Jiao Tong University Affiliated Sixth People’s Hospital. A flow diagram outlining the trial is provided in Fig. 1. This protocol was designed in accordance with the Standard Protocol Items: Recommendations for Interventional Trials (SPIRIT) guidelines [16], which can be found in Additional file 1. Adherence reminder meetings will take place before the beginning of the study. Every team member and research physician will be informed of the importance of the trial procedures and flow.

### Recruitment

Recruitment for this study began on 11 June 2017. The trial is currently actively recruiting from Shanghai Jiao Tong University Affiliated Sixth People’s Hospital, China. Eligible participants are identified and approached in the pain management centre. The patient’s consent to participate in the trial will be obtained prior to any trial-related procedures. Consent will be obtained by an appropriately trained research delegate. Patients will be asked whether they consent to the storage of their contact details so that a qualitative researcher may invite them to participate in a qualitative interview about views on SGB treatment. The qualitative researcher will collect consent for participation in this additional interview immediately prior to commencement of the interview using the qualitative interview Consent Form.

### Inclusion criteria

Participants who meet the diagnostic criteria for primary headaches [17] will be included if they are aged between 18 and 70 years and have signed an informed consent form.

### Exclusion criteria

Participants who meet any of the following criteria are not eligible for this study:

1. Participants with a space-occupying lesion
2. Known chronic liver or kidney disease
3. Participants with coagulation disorders
4. Participants with systemic or local infection
5. Participants with drug allergies
6. Psychotic patients

### Dropout criteria

Participants will be withdrawn from the study if they are not willing to continue their participation, cannot be present on the day of the experiment, or miss a treatment session and/or change their form of rehabilitation during the study.

### Intervention

All participants will undergo two SGBs, with a one-week washout period between them. For the ultrasound-guided SGB, each patient will receive 6 mL 1% lidocaine and 6 mL normal saline at one-week intervals to allow a washout period for the injection. The 1% lidocaine is composed of 2% lidocaine and normal saline in a 1:1 ratio (for example, the 1% lidocaine 2 mL is prepared by combining 2% lidocaine 1 mL, and normal saline 1 mL) [18, 19].

The patients will be positioned in a supine position with their neck slightly hyperextended. Prior to the procedure, the C7 level is confirmed by the use of a 7–14 MHz linear probe (S-Nerve, Sonosite, USA). On the short-axis view, the posterior tubercle and the vertebral body show as a slope in the transverse process of C7. Sonoanatomy of the neck at the C7 level is confirmed by the absence of the anterior tubercle. The thyroid, inferior thyroid artery, oesophagus, internal jugular vein, CCA, VA, and prevertebral fascia are also confirmed. Colour Doppler imaging is utilised to avoid penetrating the CCA, VA, and the internal jugular vein during the needle insertion. The neck area will be sterilised and the probe will be covered with sterilised vinyl. At the C7 level, the probe will be placed at the anterior scalene muscle, which is located between the carotid sheath and the brachial plexus. A 25-gauge, 6-cm needle will be inserted laterally, 5 mm from the probe. The needle tip is placed posterior to the carotid artery and anterior to the longus colli muscle under the transverse short axis for the in-plane approach. The assigned dosage of 1% lidocaine or normal saline will be injected in the patients.

At the end of the SGB, another doctor, who is not involved in the operation, will observe the ptosis and conjunctival flushing. Before and 15 min after the SGB, the flow measurements will be recorded by an ultrasound specialist. Sonograms will be obtained with a Mylab 90 (Genova, Italy) by using a linear probe (curved-array transducer, CA431, 1–8 MHz).

### Expected risks

The most serious complications of SGB include intravascular injections and retropharyngeal haematoma. The proximity of the stellate ganglion to the inferior thyroid, vertebral, or carotid arteries provides the potential for intravascular injection or vascular trauma, with resulting bleeding and haematoma [20]. Intravascular injection of even small volumes of local anaesthetic may result in loss of consciousness, apnoea, and seizure [21]. Retropharyngeal haematoma varies in severity, from mild and asymptomatic to severe and life-threatening, causing tracheal compression requiring emergency tracheotomy [22, 23]. The frequency of catastrophic retropharyngeal haematoma after SGB, with resulting airway compromise and obstruction, has been estimated as 1 in 100,000 cases [23]. Compared with the blind technique, however, ultrasound-guided SGB significantly reduced the incidence of asymptomatic haematoma [7].

Ultrasound-guided SGB, with direct visualisation of the multiple vulnerable soft tissue structures enclosed in a tight vascular space around the sympathetic chain, appears to be safer than traditional approaches. To avoid adverse risks to the patients, we will use ultrasound guidance to carefully distinguish the cervical spine, vessels, soft tissue, and trachea. We will also monitor the path and the depth of the needle in real time during ultrasound-guided SGB.

### Expected benefits

Ultrasound-guided SGB may alleviate pain in patients with primary headaches, and it may also contribute to an improved quality of life. Moreover, these participants will play an important role in correctly understanding vascular changes caused by SGB and will contribute to the scientific knowledge on the use of SGB.

### Outcomes

#### Primary outcomes

To evaluate the effect of SGB on the vascular blood flow in the neck, the primary outcomes will be the peak systolic velocity (PSV), the end diastolic velocity (EDV), diameter, and resistance index (RI) of the CCA and VA at 15 min after the first SGB (T1). Sonograms will be obtained with a Mylab 90 (Genova, Italy) by using a 4–10-MHz linear transducer. For flow-volume measurements, a straight segment of the common carotid artery at least 2 cm below the carotid bulb will be selected [24].

All measurements will be performed by an ultrasound specialist using the same ultrasound instrument.

#### Secondary outcomes

Secondary outcomes include: (1) the PSV, EDV, RI, and diameter of the CCA and VA at T0, T2, T3, and T4; (2) the rate of ptosis and conjunctival flushing at T1 and T3 (ptosis and conjunctival flushing are considered the standard indicators of SGB of success [25]); (3) the numerical rating scale (NRS) at T0 and T4. The NRS allows the patient to describe the intensity of his/her pain as a number, usually ranging from 0 to 10, where 0 means no pain and 10 means pain as “bad as it could be” [26].

**Fig. 2:**
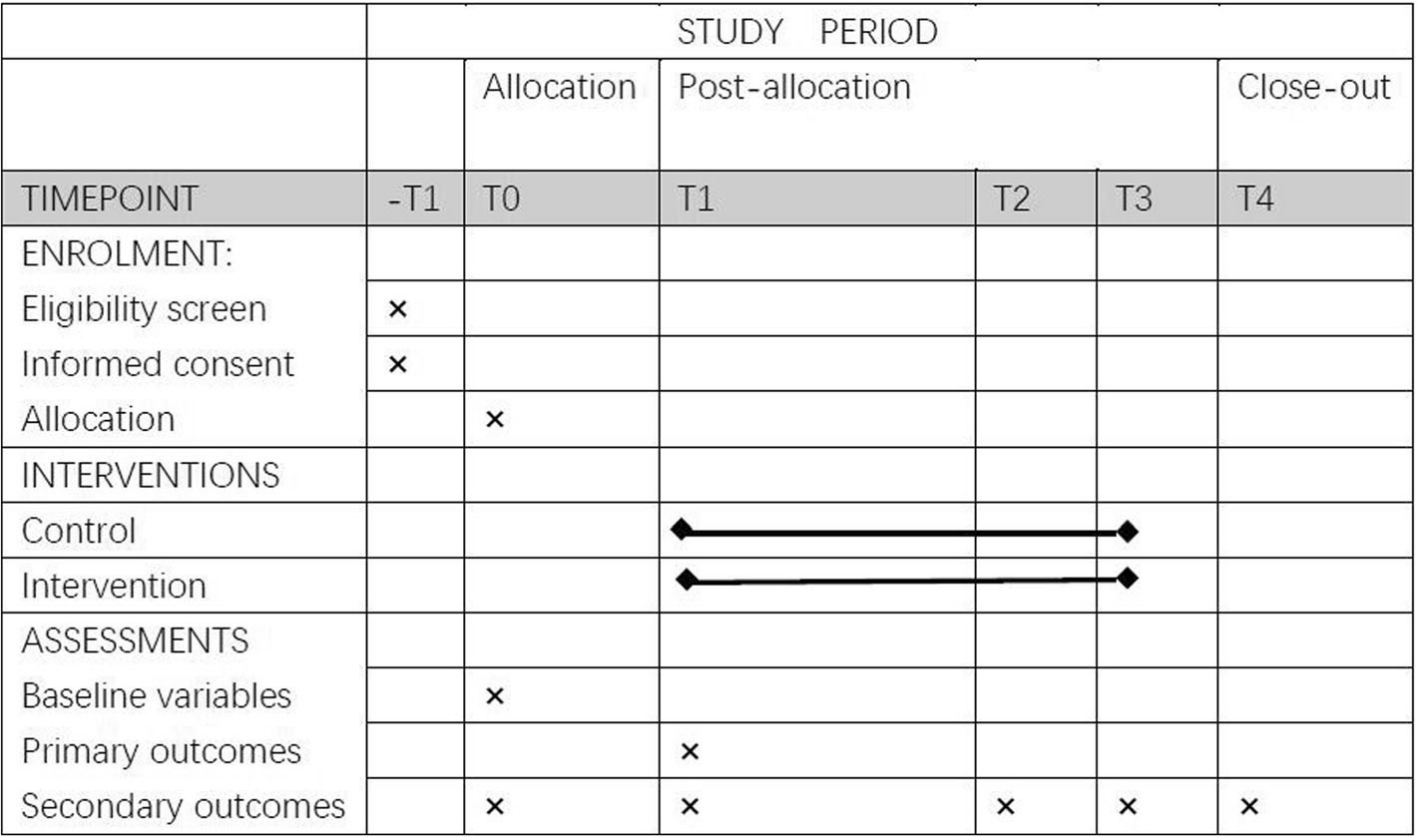
Schedule of enrolment, interventions, and assessments. SPIRIT 2013 recommended content for the schedule of enrolment, interventions, and assessments

Adverse events (AEs) or serious adverse events (SAEs) will be collected at each assessment time point and any AEs or SAEs related to the intervention will be reported. For an overview of the schedule of enrolment, interventions, and assessments please see Fig. 2.

### Sample size

The sample size was calculated based on the CCA blood flow results reported previously [11]. With an expected difference of 10 cm/s in peak systolic velocity of the CCA between group means, an SD of 10 cm/s, α = 0.05, and β = 0.8, a sample size of 17 patients was required in each group. To compensate for a dropout rate of up to 10%, we will enrol a total of 38 cases (19 for each group) for this study. Although the study is powered to allow a 15% dropout rate, we will make every effort to minimise patient loss and missing data during the trial period.

### Randomisation

Participants will be randomly allocated to either group 1 or group 2 with a 1:1 allocation defined by a computer-generated randomisation using the R package (R Foundation for Statistical Computing). The random allocation sequence will be generated by a statistical expert. Randomisation will be under the control of a single investigator who will be the only person allowed to manage the electronically secured file containing the subject randomisation assignments. This investigator will be blinded to the group to which the participants are allocated.

### Blinding

The participants, researchers, and outcome assessors will remain blind to group allocation throughout the study. Participants will be assigned codes and will be concealed during the allocation process to ensure proper blinding. The researchers responsible for performing the intervention and evaluating the outcomes will not know the study design, allocation, objectives, or expected outcomes.

### Data collection process

We will confirm eligibility criteria are met through review of the participant’s medical records. The following data will be collected for each participant: patient identifiers and demographic information, study intervention details, and the peak systolic velocity, vessel diameter, and RI of the CCA and VA, as measured at T0, T1, T2, T3, and T4. The rate of ptosis and conjunctival flushing will be measured at T1 and T3; the pain scores will also be recorded at T0 and T4.

### Monitoring of data quality

The peak systolic velocity, vessel diameter, and RI of the CCA and VA will be detected by an ultrasound specialist using the same ultrasound instrument. The rate of ptosis and conjunctival flushing will be recorded by a doctor. Another pain physician will teach patients to use the NRS scoring system and then record pain scores. Data are directly recorded in the electronic study database, which is backed up regularly. All of the assessors will be blinded from the information linking participants and interventions. Study participation will last 3 weeks from enrolment for each participant. No interim analysis will be performed during the study.

### Statistical analyses

Baseline demographic characteristics, including individual variables such as age, sex, weight, and other baseline values, will be expressed with descriptive statistics for the two groups. We will use the SPSS Statistics V. 21.0 (SPSS-IBM®) to perform all statistical analyses. The normality and homogeneity of all variable distributions will be tested with the Shapiro-Wilk test and Levene’s test, respectively. For nonparametric data, the Friedman test will be used followed by the Wilcoxon post hoc test. We will use two-way (2 × 4) ANOVA for inferential statistical analysis of the parametric data, with Tukey’s post hoc tests. All data will be represented by the mean ± standard deviation. Significance level will be set as α ≤ 0.05.

The main analysis will be performed after all patients have completed the study. A statistical analysis plan will be written before the data are analysed.

## Discussion

The purpose of this study was to investigate the effect of SGB on cervical blood flow and to observe the efficacy of SGB in patients with primary headaches.

Ultrasound techniques are useful both in measurement of the cervical vascular [27, 28] and SGB [6, 29, 30]. Cervical sympathetic and SGBs provide a valuable diagnostic and therapeutic benefit to sympathetically maintain pain syndromes in the head. Identifying the correct fascial plane can be achieved with ultrasound guidance, thus facilitating the caudal spread of the injectate to reach the stellate ganglion at C7 level. This allows for a more effective and precise sympathetic block with the use of a small injectate volume. Accordingly, the risk of vascular and soft tissue injury may be minimised [6].

According to International Classification of Headache Disorders, second edition (ICHD-2)-based studies, primary headaches corresponded to 50.1–78.4% of headaches and 2.5–23% of the cases were unclassified [31, 32]. Primary headaches have complex pathophysiologies, and the exact mechanism of the disease is incompletely understood [33]. Therefore, treatment of the primary headaches is also complicated. Blocking the sympathetic nerve aborts an acute attack of cluster headaches and may play a major role in aborting the cluster [34]. We will select patients with primary headache to participate in this study and expect that SGB will alleviate the pain in these patients.

SGB can cause changes in vascular blood flow in the head and neck, but research in this area is limited and often controversial [9–12]. We expect the outcomes of the present study to help us to understand the connections between the cervical sympathetic nervous system and blood vessels and provide additional clinical evidence of the potential benefits of SGB as applied to primary headaches.

### Trial status

Enrolment began on 11 July 2017. As of 12 October 2017, we have enrolled 27 of our target 38 participants in the study. It is expected that recruitment will be completed by December 2017.

## Additional file

Additional file 1: SPIRIT (Standard Protocol Items: Recommendations for Interventional Trials). Completed SPIRIT 2013 checklist of recommended items to address in a clinical trial protocol and related documents. (DOC 123 kb)

## Abbreviations

AEs: Adverse events
BA: Arteriae basilaris
C7: Seventh cervical
CCA: Common carotid artery
ChiCTR: Chinese Clinical Trial Registry
CONSORT: Consolidated Standards of Reporting Trials
EDV: End diastolic velocity
ICA: Internal carotid artery
ICHD-2: International Classification of Headache Disorders, second edition
ION: Ischaemic optic neuropathy
MCA: Middle cerebral artery
NRS: Numerical rating scale
PSV: Peak systolic velocity
RI: Resistance index
SAE: Serious adverse event
SAH: Subarachnoid haemorrhage
SGB: Stellate ganglion block
SPIRIT: Standard Protocol Items: Recommendations for Interventional Trials
VA: Vertebral artery
